# Enhanced Chemo‐Immunotherapy Strategy Utilizing Injectable Thermosensitive Hydrogel for The Treatment of Diffuse Peritoneal Metastasis in Advanced Colorectal Cancer

**DOI:** 10.1002/advs.202303819

**Published:** 2023-10-24

**Authors:** Meng Wang, DanRong Hu, Yun Yang, Kun Shi, JiaNan Li, QingYa Liu, YiCong Li, Ran Li, Meng Pan, Dong Mo, Wen Chen, XiCheng Li, ZhiYong Qian

**Affiliations:** ^1^ Department of Biotherapy Cancer Center and State Key Laboratory of Biotherapy West China Hospital Sichuan University Chengdu 610041 China; ^2^ Rehabilitation Medicine Center and Institute of Rehabilitation Medicine Key Laboratory of Rehabilitation Medicine in Sichuan Province West China Hospital Sichuan University Chengdu 610041 China

**Keywords:** chemo‐immunotherapy, colorectal cancer, diffuse peritoneal metastasis, intraperitoneal chemotherapy, thermosensitive hydrogel

## Abstract

Patients with colorectal cancer (CRC) and diffuse peritoneal metastasis (PM) are not eligible for surgical intervention. Thus, palliative treatment remains the standard of care in clinical practice. Systemic chemotherapy fails to cause drug accumulation at the lesion sites, while intraperitoneal chemotherapy (IPC) is limited by high clearance rates and associated complications. Given the poor prognosis, a customized OxP/R848@PLEL hydrogel delivery system has been devised to improve the clinical benefit of advanced CRC with diffuse PM. This system is distinguished by its simplicity, security, and efficiency. Specifically, the PLEL hydrogel exhibits excellent injectability and thermosensitivity, enabling the formation of drug depots within the abdominal cavity, rendering it an optimal carrier for IPC. Oxaliplatin (OxP), a first‐line drug for advanced CRC, is cytotoxic and enhances the immunogenicity of tumors by inducing immunogenic cell death. Furthermore, OxP and resiquimod (R848) synergistically enhance the maturation of dendritic cells, promote the expansion of cytotoxic T lymphocytes, and induce the formation of central memory T cells. Moreover, R848 domesticates macrophages to an anti‐tumor phenotype. OxP/R848@PLEL effectively eradicates peritoneal metastases, completely inhibits ascites production, and significantly prolongs mice lifespan. As such, it provides a promising approach to managing diffuse PM in patients with CRC without surgical indications.

## Graphical abstract

Patients with colorectal cancer (CRC) and diffuse peritoneal metastasis (PM) are not eligible for surgery. A customized OxP/R848@PLEL hydrogel delivery system has been developed. The PLEL hydrogel forms a drug depot within the abdominal cavity. OxP can enhance the immunogenicity of tumors. OxP/R848 exhibits a “tumor vaccine‐like” effect that elicits specific immunity. OxP/R848@PLEL effectively eradicates PM and significantly prolongs mice lifespan.

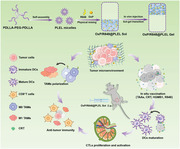

## 1 Introduction

Colorectal cancer (CRC) is the most prevalent malignant tumor of the digestive system and is characterized by high morbidity and mortality rates.^[^ ^1^ ^]^ The low early diagnosis rate of CRC can be attributed to ambiguous abdominal symptoms and a lack of endoscopic resources.^[^ ^2^ ^]^ At the initial diagnosis, distant metastasis is present in 44% of patients. Peritoneal metastases (PM) accounts for 25% of all cases.^[^ ^3^ ^]^ PM is the most intractable form of metastasis, resulting in carcinogenic consumption, intestinal obstruction, and cancerous ascites.^[^ ^4^ ^]^ Cancerous ascites develop rapidly, leading to abdominal distension, pain, poor appetite, and other symptoms that substantially impair patients' quality of life.^[^ ^5^ ^]^ Alarmingly, the median overall survival (mOS) of patients with CRC and PM is 16.3 months, whereas the mOS of patients with malignant ascites does not exceed 4 months.^[^ ^6^, ^7^, ^8^ ^]^

For patients diffuse PM, surgery fails to prolong patient survival and poses a risk of complications, such as enterocutaneous fistula, wound infection, and wound dehiscence.^[^ ^9^, ^10^ ^]^ In this scenario, palliative systemic chemotherapy is adopted to maximize survival.^[^ ^11^, ^12^ ^]^ However, the dosage of systemic chemotherapy is restricted by toxicity and the patient's physical condition, resulting in suboptimal drug concentrations at tumor sites and mediocre therapeutic outcomes.^[^ ^13^, ^14^ ^]^ Thus, intraperitoneal chemotherapy (IPC) has attracted attention from oncology researchers due to its ability to increase drug concentration in abdominal lesions and reduce the incidence of systemic adverse reactions.^[^ ^15^ ^]^ Unfortunately, IPC has certain limitations, including limited drug retention time, uneven distribution of drugs, and potential complications such as bleeding and intestinal perforation.^[^ ^16^, ^17^ ^]^ Therefore, developing novel drug formulations that can effectively eradicate PM and suppress ascitic fluid production is imperative.

Hydrogels with biocompatibility and tunable mechanical properties may present an alternative solution for overcoming the limitations of IPC.^[^ ^18^, ^19^, ^20^ ^]^ Compared to nanocarriers, hydrogels made from suitable materials are biodegradable and less toxic, making them crucial in drug delivery, 3D cell culture, tissue engineering, and other medical fields. Hydrogels can be customized to facilitate subcutaneous, rectal, and intraperitoneal delivery according to pathological characteristics, indicating their potential use in localized drug delivery for a variety of solid tumors.^[^ ^21^, ^22^ ^]^ Furthermore, intelligent hydrogels can detect and react to environmental stimuli for precise drug delivery.^[^ ^23^, ^24^, ^25^ ^]^ Due to its excellent biocompatibility, facile modifiability, and high drug encapsulation efficiency, extensive research and development have been conducted on PEG‐based hydrogels.^[^ ^26^ ^]^ In our previous study, we successfully synthesized a biodegradable PDLLA‐PEG‐PDLLA (PLEL) hydrogel with an appropriate phase‐transition temperature.^[^ ^27^ ^]^ The PLEL hydrogel exhibits a flowable sol state at room temperature (RT), facilitating drug loading and injection. After in vivo administration, it undergoes a phase transition to form a drug‐loaded gel depot for sustained drug release.^[^ ^28^ ^]^ Moreover, PLEL hydrogels can ensure the uniform distribution of drugs in the abdominal cavity and effectively prolong drug retention time. Their injectability and biodegradability considerably reduce complications, thereby improving patient compliance.

In addition to chemotherapy, immunotherapy has brought possibilities for treating refractory cancers.^[^ ^29^ ^]^ Oxaliplatin (OxP) is the preferred first‐line agent for treating advanced metastatic CRC.^[^ ^30^ ^]^ Apart from to cytotoxicity, OxP can induce immunogenic cell death (ICD), which promotes the release of damage‐associated molecular patterns, boosts exposure to tumor‐associated antigens (TAAs), and thus improves tumor immunogenicity.^[^ ^31^, ^32^ ^]^ However, the immunity triggered by ICD effects is insufficiently robust in most cases.^[^ ^33^ ^]^ With advancements in vaccines, immune adjuvants have become essential components of cancer vaccines.^[^ ^34^ ^]^ As the most capable antigen‐presenting cells (APCs), the maturation of dendritic cells (DCs) is crucial for initiating anti‐tumor immunity.^[^ ^35^ ^]^ The immune adjuvant resiquimod (R848) can stimulate toll‐like receptor 7/8 (TLR7/8) and promote DCs maturation, thus inducing cytotoxic T lymphocytes (CTLs) response.^[^ ^36^ ^]^ Furthermore, R848 polarizes tumor‐associated macrophages (TAMs) into pro‐inflammatory phenotypes (M1), alleviating the immunosuppressed tumor microenvironment (TME).^[^ ^37^ ^]^ Therefore, OxP combined with R848 exhibits a “tumor vaccine‐like” effect that elicits specific immunity and produces an efficient and long‐lasting anti‐tumor response.

**Figure 1:**
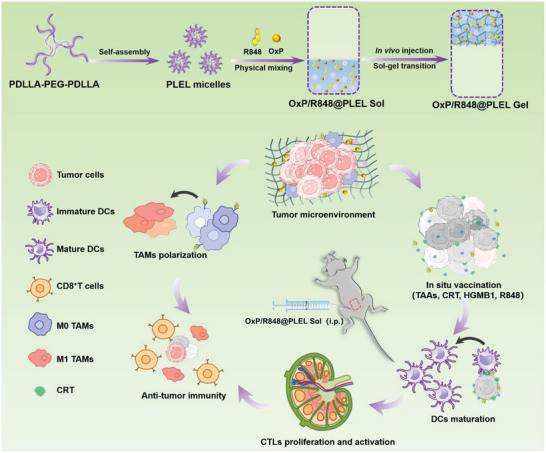
Schematic illustration of the preparation and mechanism of OxP/R848@PLEL injectable thermosensitive hydrogel, which exerts synergetic chemo‐immunotherapy in the treatment of advanced CRC with diffuse PM, through induction of ICD, maturation of DCs, and polarization of TAMs.

Herein, we further explored the potential benefits of a chemo‐immunotherapy strategy based on thermosensitive hydrogels. We fabricated an OxP/R848@PLEL drug‐loading system via simple physical mixing techniques (**Figure** **1**, scheme). Subsequently, we evaluated the thermosensitivity and sustained‐release ability of the PLEL hydrogel. Furthermore, the synergistic effects of OxP and R848 were explored by examining cell proliferation, ICD induction, DCs maturation, and macrophages polarization. Finally, a pharmacodynamic experiment was conducted to assess the anti‐tumor efficacy of OxP/R848@PLEL in terms of PM, ascites volume, and immune memory formation. This study will provide innovative insights into managing of CRC with inoperable PM.

## 2 Results and Discussion

### 2.1 Preparation and Characterization of OxP/R848@PLEL

We synthesized PLEL block copolymers via the ring‐opening polymerization of polyethylene glycol (PEG) and D, L‐lactide (D, L‐LA) catalyzed by stannous octoate (Sn(Oct)_2_). The structure and molecular weight were characterized by ^1^H NMR spectroscopy (Figure S1A, Supporting Information) and gel permeation chromatography (Figure S1B, Supporting Information), respectively. Subsequently, we prepared OxP/R848@PLEL by simple physical mixing of OxP and R848 with the PLEL micelle solution at 25 °C at a certain ratio (OxP: 0.6 mg mL^−1^, R848: 0.1 mg mL^−1^).

**Figure 2:**
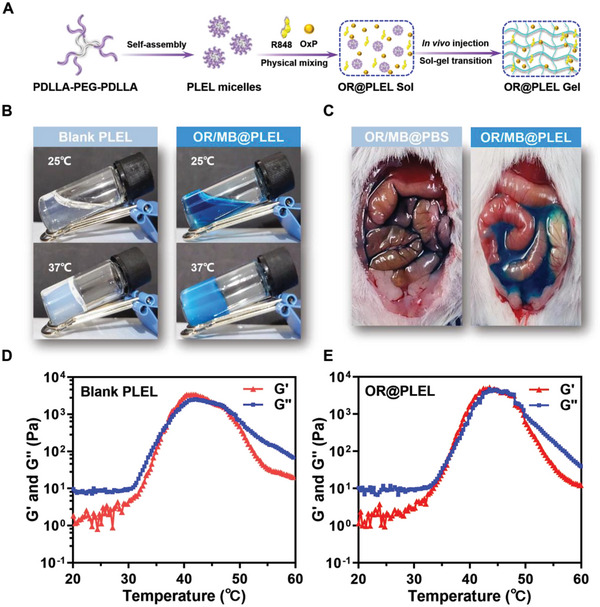
The properties of PLEL micelles. A) Diagram illustrating the preparation and gelation of OR@PLEL. B) In vitro sol‐gel transition of blank gel (20 wt%) and OR/MB@PLEL (OxP, R848, methylene blue). C) Photographs of OR/MB@PBS and OR/MB@PLEL in vivo. D,E) Variations in storage modulus (G') and loss modulus (G”) of blank PLEL (20 wt%) and OR@PLEL as a function of temperature.

PLEL is an amphiphilic block copolymer consisting of hydrophilic PEG segment and hydrophobic polylactic acid segment, which can spontaneously form micelles with a shell‐core structure in an aqueous solution (**Figure** **2**A). The suitability of PLEL as an IPC carrier depends on its ability to undergo sol‐gel transitions at physiological temperatures.^[^ ^38^ ^]^ The in vitro gelation of OxP/R848@PLEL was assessed using the tube inversion method (Figure 2B). Methylene blue was additionally incorporated into the gels to prevent confusion. The system exhibited a flowable sol state at 25 °C, which presents advantages for drug loading and injection. With the temperature increase, PLEL micelles became enlarged, leading to aggregation and bridging (Figure S1C, Supporting Information), forming a semisolid hydrogel at 37 °C. The gelation ability was observed for the drug‐containing gel after administration (Figure 2C).

To further elucidate the dynamic mechanical behavior of the PLEL hydrogel during temperature variations, dynamic rheology experiments were conducted (Figure 2D,E). Consistent with the macroscopic characterization, OxP/R848@PLEL exhibited thermosensitive behavior comparable to that of blank PLEL. Specifically, when the temperature exceeded 30 °C, both the storage modulus (G’) and the loss modulus(G’’) exhibited a rapid increase, and the G’’ was surpassed ≈37 °C, which indicated that the bulk phase of the system tends to behave as a viscoelastic solid. Additionally, the system maintained a gel state within the range of 37 to 42 °C, demonstrating exceptional compatibility and adaptability with body temperature. These results suggested that the PLEL hydrogel possesses both injectability and in vivo gelation ability, rendering it a promising candidate for IPC.

### 2.2 Drug Release Performance In Vitro and In Vivo

**Figure 3:**
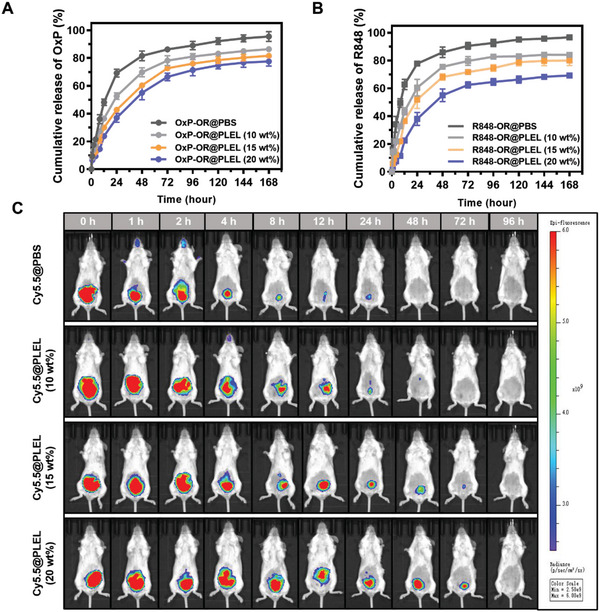
In vitro release and in vivo retention of drug‐loaded PLEL hydrogel. A,B) Release behavior of OxP and R848 in PBS or different PLEL micelles concentrations (n = 3). C) In vivo fluorescence images of Cy5.5 captured by IVIS in different release media. (n = 3). Data are presented as mean ± SD.

The efficacy of chemotherapeutic agents is directly proportional to their concentration and duration of exposure at the target site. IPC can directly improve drug concentration at the abdominal tumor site. However, the rapid absorption of small molecule drugs by the portal vein circulation limits its efficacy.^[^ ^39^ ^]^ Therefore, it is imperative for IPC delivery carriers to possess good sustained‐release ability. An improved dialysis method was employed to investigate the in vitro release behavior of OxP (**Figure** **3**A) and R848 (Figure 3B) from PLEL hydrogels. Within 24 h, ≈70% of the free OxP was released, while only 37.3% was released with 20 wt% PLEL. After 24 h, ≈80% of the free R848 was released, whereas the release in 20 wt% PLEL was less than half of that observed for the free drug. Overall, free OxP and R848 exhibited a cumulative release of ≈90% within 72 h, whereas a substantial retardation in drug release was observed with the PLEL hydrogel.

Compared with the simple and easily controllable in vitro environment, the in vivo physiological milieu is more complex. The mechanical properties of hydrogels are influenced by intraperitoneal gastrointestinal peristalsis, hydrolysis, and enzymatic catalysis.^[^ ^40^ ^]^ To evaluate the drug retention capacity of PLEL hydrogel in vivo, we used Cy5.5 a model drug and monitored the fluorescence intensity changes using the IVIS Lumina III imaging system (Figure 3C). The in vivo release of Cy5.5 (Figure S2B, Supporting Information) from the different formations was quantified according to the fluorescence intensity (Figure S2A, Supporting Information). In the PBS group, the fluorescence intensity rapidly declined after injection, with an 87.9% release within 24 h. Conversely, the 20 wt% PLEL hydrogel exhibited sustained drug release efficacy in vivo for up to 4 days. Specifically, the amount release at 72 h (81.5%) was lower than that released at 24 h.

Drug retention was prolonged with increasing concentrations of PLEL, but the high viscosity of PLEL at concentrations above 20 wt% impeded filtration sterilization and intraperitoneal administration. Considering the sustained‐release ability of PLEL and the operability of the treatment, the 20 wt% PLEL hydrogel was selected as the optimal drug delivery carrier for IPC in subsequent experiments. Based on the pharmacokinetics of the drug, a 4‐day administration cycle was employed for anti‐tumor therapy in vivo.

### 2.3 Cytotoxicity Assay

**Figure 4:**
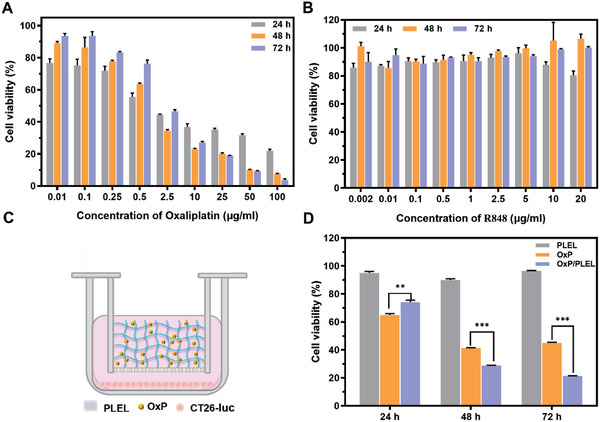
In vitro cell viability of CT26‐luc treated with different formations. A,B) Viability of CT26‐luc cells treated with varying concentrations of OxP or R848 in vitro (n = 3). C) Schematic diagram depicting the construction of a transwell system for co‐incubation of OxP@PLEL and CT26‐luc cells. D) Cell viability of CT26‐luc co‐incubated with blank PLEL, free OxP, or OxP@PLEL for 24, 48, and 72 h (n = 3). Data are presented as mean ± SD. *P* values were determined by Student's t‐test (**p < 0.01, ***p < 0.001).

Platinum‐based anticancer agents can effectively impede DNA synthesis by forming intra‐ and inter‐strand cross‐links with DNA, inhibiting of cell proliferation.^[^ ^41^ ^]^ To assess the cytotoxicity of OxP on CT26‐luc cells, a CCK‐8 assay was performed (**Figure** **4**A). Overall, the viability of CT26‐luc cells decreased in a dose‐dependent manner with increasing OxP concentrations. The relative activity of cells treated with OxP at concentrations below 10 µg mL^−1^ increased gradually over time. This implies that a threshold of OxP dosage must be surpassed to effectively inhibit tumor cell proliferation. In addition, R848‐treated CT26‐luc cells exhibited viability of over 80% (Figure 4B), and negligible cytotoxicity.

Furthermore, using hydrogel as a carrier for IPC enhances the efficacy of chemotherapy drugs by extending their duration of action. To investigate the impact of sustained release of PLEL hydrogel on OxP efficacy, a transwell co‐culture system was set up to evaluate the cytotoxicity of OxP@PLEL (Figure 4C). In the experiment, we replaced the original medium with a fresh medium every 24 h to simulate in vivo drug clearance. The cell viability was measured at 24, 48, and 72 h, respectively, and the results are illustrated in Figure 4D. In the first 24 h, a portion of the OxP in the OxP@PLEL group was sequestered in the gel matrix, resulting in reduced cytotoxicity. Following the medium exchange, OxP in the control group was eliminated, whereas it was continuously released from the OxP@PLEL group. Consequently, the cytotoxicity of the OxP@PLEL group was considerably higher than that of the control group at 48 and 72 h. PLEL exhibited minimal cytotoxicity toward CT26‐luc cells, with a cell survival rate exceeding 90%. These findings suggest that the PLEL hydrogel may augment the anti‐tumor efficacy of OxP by retarding its clearance.

### 2.4 Immunological Analysis In Vitro

**Figure 5:**
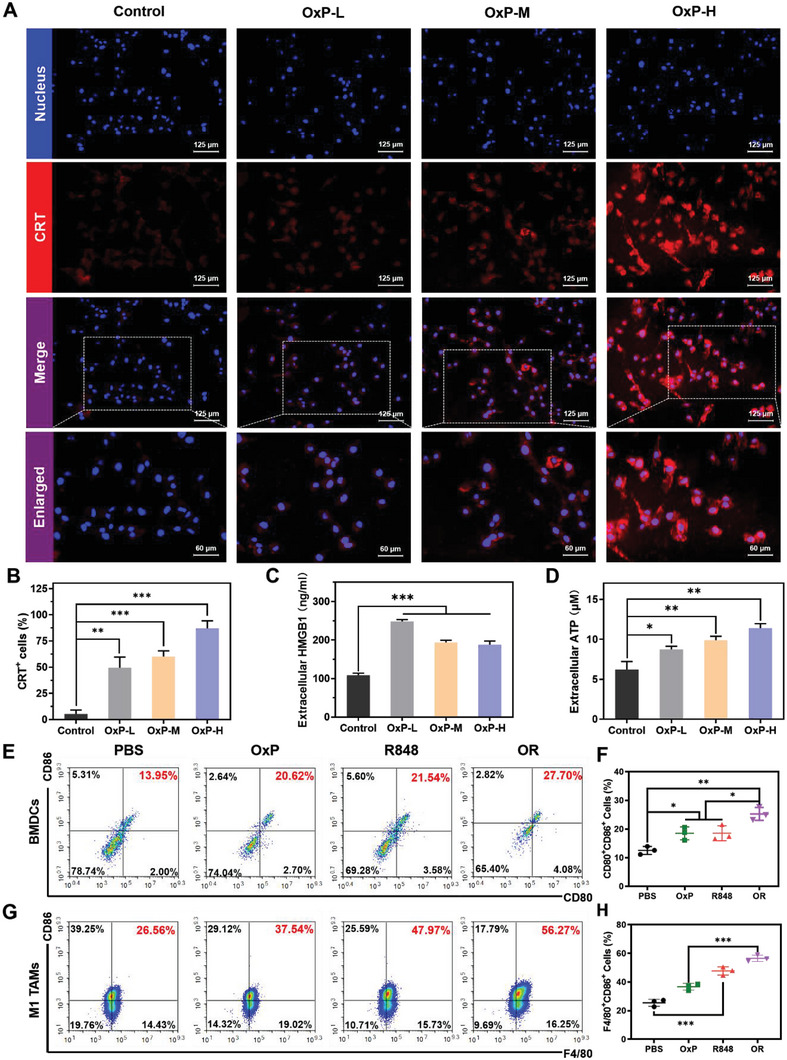
Immunological analysis of OxP and R848. A,B) Immunofluorescence of CRT and the percentage of CRT^+^ cells (n = 3). C,D) Content of HMGB1 and ATP in culture supernatant (n = 3). E,F) Flow cytometry of CD80 and CD86 on the BMDCs (n = 3). G,H) Proportion of M1 phenotype Raw264.7 cells after different treatments (n = 3). Data are presented as mean ± SD. *P* values were determined by Student's *t*‐test and one‐way ANOVA with Tukey's test (*p < 0.05, **p < 0.01, ***p < 0.001).

Cytotoxic drugs, such as OxP, can induce ICD and elicit specific anti‐tumor immunity.^[^ ^42^ ^]^ ICD is crucial in improving the efficacy of chemotherapy and eradicating small tumor foci.^[^ ^43^ ^]^ OxP induces endoplasmic reticulum stress, exposing calreticulin (CRT) to the membrane and facilitating antigen uptake by DCs.^[^ ^44^ ^]^ Using immunofluorescence, we detected CRT expression in CT26‐luc cells treated with low, medium, and high concentrations of OxP. As depicted in **Figure** **5**A,B, CRT‐positive cells were scarcely detected in the PBS group. In contrast, red fluorescence was observed in the OxP group, and the fluorescence intensity was dose‐dependent. The proportion of CRT‐positive cells exceeded 80% at high OxP doses. During ICD, high mobility group protein B1 (HMGB1) released from dying cells activates toll‐like receptor 4 (TLR4) and promotes the secretion of inflammatory cytokines.^[^ ^45^ ^]^ Additionally, ATP can recruit immature DCs and macrophages to ICD sites.^[^ ^46^ ^]^ Subsequently, the concentrations of both HMGB1 and ATP in the supernatant were measured using ELISA (Figure 5C,D). OxP promoted the release of HMGB1 and ATP from CT26‐luc cells. By detecting the expression of ICD marker molecules, including CRT, HMGB1, and ATP, we demonstrated that OxP enhanced the immunogenicity of tumor sites by inducing ICD.

DCs are the most potent APCs, and can stimulate initial T‐cell activation and proliferation.^[^ ^47^ ^]^ Therefore, DCs maturity is a critical factor for inducing specific anti‐tumor immunity.^[^ ^48^ ^]^ HMGB1 and R848 promote DCs maturation by activating TLR4 and TLR7/8, respectively. We established a co‐culture system to investigate the effects of OxP‐treated CT26‐luc cells and R848 on DCs maturation (Figure 5E,F). CD80 and CD86 are important co‐stimulatory molecules that serve as markers of DCs maturity. The results revealed that OxP and R848 alone up‐regulated the expression of CD80 and CD86 in CD11c‐positive cells. Furthermore, the combination of these two agents synergistically enhanced the bone marrow‐derived dendritic cells (BMDCs) maturity. TAMs are among the most abundant immune cells in CRC.^[^ ^49^ ^]^ TAMs can be domesticated by the TME to facilitate tumor progression and metastasis.^[^ ^50^ ^]^ R848 can polarize them into an anti‐tumor M1 phenotype by activating the Nuclear Factor kappa B (NF‐κB) pathway.^[^ ^51^ ^]^ This study yielded the anticipated flow cytometry results verified that R848 alone or combined with OxP could polarize Raw264.7 cells to the M1 phenotype (Figure 5G,H). Additionally, we confirmed the tumor killing effect of M1‐TAMs through a transwell co‐culture assay (Figure S3C,D, Supporting Information).

### 2.5 Dose Optimization of OxP/R848@PLEL In Vivo

**Figure 6:**
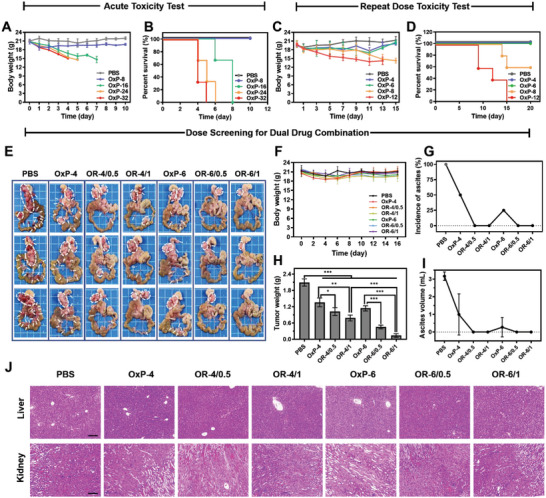
Dose optimization of OxP/R848@PLEL in PM treatment. A,B) Changes of body weight and survival of mice in OxP@PLEL acute toxicity test (n = 3). C,D) Changes in body weight and survival of mice in OxP@PLEL repeated administration toxicity test (n = 5). E) Photographs of PM in dose screening for dual combination. F–I) Changes in body weight, tumor weight, the incidence of ascites, and ascites volume (n = 4). J) H&E staining of liver and kidney tissues of each group. Scale bars: 200 µm. Data are presented as mean ± SD. *P* values were determined by Student's *t*‐test and one‐way ANOVA with Tukey's test (*p < 0.05, **p < 0.01, ***p < 0.001).

The anti‐tumor efficacy of cytotoxic chemotherapeutic drugs is positively correlated with their dosage. However, excessive dosing may result in severe adverse reactions and fatalities. To ensure safety, an acute toxicity test was initially performed by administering a single dose of OxP@PLEL to mice and monitoring changes in body weight and mortality (**Figure** **6**A,B). Mice administered a dose of 16 mg kg^−1^ or higher exhibited a continuous decline in body weight and eventually died within 8 days, whereas those in the 8 mg kg^−1^ group initially experienced a decrease but gradually returned to baseline levels. The findings revealed that sole administration of OxP@PLEL at 8 mg kg^−1^ was well tolerated by the mice.

Subsequently, we conducted a repeated administration experiment with three doses administered at 4‐day intervals, considering the retention time of the drug and tumor growth (Figure 6C,D). However, none of the mice treated with 12 mg kg^−1^ survived. Mice administered 8 mg kg^−1^ began to die after the third dose, resulting in a survival rate of 60%. Conversely, all mice appeared normal and survived at doses of 4 or 6 mg kg^−1^. Based on these results, a dosage of 4 or 6 mg kg^−1^ OxP@PLEL may be used to screen anti‐tumor efficacy.

Given the diverse range available, reference standards for the effective doses of combination chemotherapy drugs and immune adjuvants are lacking. Furthermore, it remains unclear whether the combination of OxP and R848 exacerbates OxP toxicity. Therefore, we optimized the dose of OxP/R848@PLEL to achieve maximum anti‐tumor efficacy while minimizing adverse effects. Regarding toxicity, the mice in each group displayed good vital signs and maintained a stable body weight throughout the study period (Figure 6F). No pathological changes were observed in the major organs (Figure 6J; Figure S4, Supporting Information). Regarding the therapeutic effect (Figure 6E,H–I), OxP@PLEL alone exhibited a degree of therapeutic effect against PM. However, multiple metastatic foci and ascites persisted. In contrast, OxP/R848@PLEL reduced PM and inhibited ascites production. The optimal anti‐tumor effect (inhibition rate: 91.6%) was achieved with OxP/R848 at doses of 6 and 1 mg kg^−1^. Based on their safety and efficacy, these doses were used for subsequent anti‐tumor pharmacodynamic investigations.

### 2.6 Anti‐Tumor Effect of OxP/R848@PLEL in Peritoneal Metastasis Model

**Figure 7:**
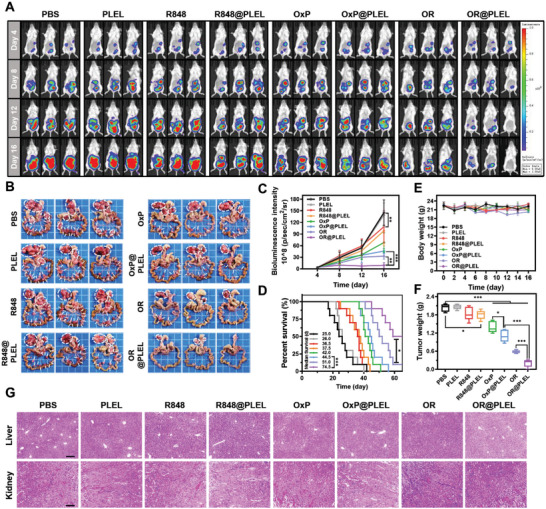
In vivo pharmacodynamic study of OxP/R848@PLEL in PM treatment. A,C) In vivo image of fluorescence and quantitative analysis of fluorescence intensity (n = 5). B) Photographs of PM foci of CRC. D) Survival condition of mice after various treatment (n = 10). *P* values were determined by Gehan‐Breslow‐Wilcoxon test (*p < 0.05, ***p < 0.001). E,F) Body weight and tumor weight of mice in each group (n = 5). G) Pathological sections of the liver and kidney. Scale bars: 200 µm. Data are presented as mean ± SD. *P* values were determined by Student's *t*‐test and one‐way ANOVA with Tukey's test (*p < 0.05, **p < 0.01, ***p < 0.001).

Following the determination of a safe and effective dose, pharmacodynamic experiments were conducted to investigate the role of each component in OxP/R848@PLEL. To dynamically evaluate tumor growth, IVIS was performed every 4 days to monitor tumor occurrence and development (**Figure** **7**A). On the fourth day, oval spots with distinct boundaries were detected in the abdomen of the mice, indicating successful colonization and formation of multiple metastatic foci by tumor cells. A rapid increase in fluorescence intensity was observed in the control group over time. On day 16, widespread tumor metastases were observed in the gastric ligament and mesentery of the control group (Figure 7B), indicating the successful establishment of an advanced diffuse PM model for CRC.

The anti‐tumor effect was comprehensively evaluated by dissecting the posterior gastric ligament area and mesenteric tumor nodules (Figure 7B), quantitative analysis of fluorescence intensity of in vivo imaging (Figure 7C), measuring the tumor weight (Figure 7F), and assessing the survival time (Figure 7D). The body weights of the mice in the PLEL group were normal, and no pathological damage was observed in the major organs (Figure 7G; Figure S5A, Supporting Information), indicating the excellent biocompatibility of the PLEL hydrogel. PLEL delivery of OxP/R848 resulted in a higher tumor inhibition rate (90.0% vs 71.2%), while OxP and R848 also increased inhibition rates compared with their respective free drug groups (45.6% vs 31.3%, and 11.2% vs 9.16%, respectively). This suggests that the sustained release effect of the PLEL hydrogel can prolong the drug action time and enhance anti‐tumor efficacy.

In patients with CRC, PM onset signifies the rapid progression of the disease. OxP is the preferred first‐line treatment for patients with advanced metastatic CRC.^[^ ^52^ ^]^ Based on the fluorescence intensity trend, although the OxP monotherapy group exhibited lower fluorescence intensity than the model group, it only delayed tumor progression. The tumor‐inhibitory effect of R848 was negligible, possibly because of its lack of tumor specificity in inducing immune responses. Hopefully, the remarkable anti‐tumor efficacy of OxP/R848@ PLEL was demonstrated by its low tumor burden and a 50% survival rate beyond 60 days. In addition, these mice did not develop ascites (Figure S5B, Supporting Information), which substantially alleviated their discomfort.

### 2.7 Anti‐Tumor Immune Memory Induced by OxP/R848@PLEL

Tumor recurrence poses challenges to tumor treatment. Clinical practice has revealed that all visible tumor lesions can be eliminated through treatment; however, even small undetectable residual lesions may lead to tumor recurrence.^[^ ^53^ ^]^ The physical condition of patients often deteriorates after the initial treatment, and achieving the desired effect in subsequent treatments is difficult.^[^ ^54^ ^]^ Therefore, immune memory formation in tumors after initial treatment can effectively prevent tumor recurrence.

**Figure 8:**
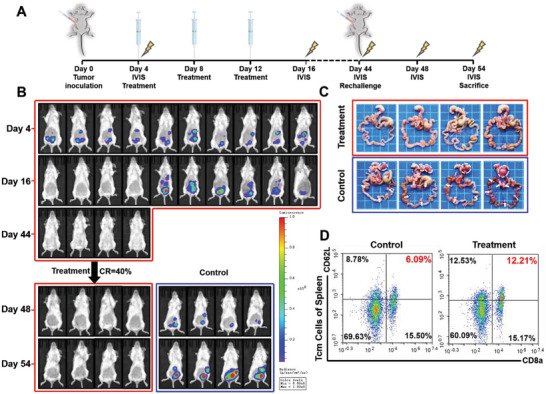
Anti‐tumor immune memory induced by OxP/R848@PLEL. A) Time node of drug administration, imaging and rechallenge. B) Fluorescence images of IVIS (n = 10 in treatment group, n = 4 in control group). C) Photographs of PM foci of CRC on day 54. D) Flow cytometry of central memory T cells in the spleen (n = 3).

Encouraging by the result that OxP/R848@PLEL achieved complete tumor remission in some mice, we further examined whether these mice could develop effective anti‐tumor immune memory (**Figure** **8**A). We initially administered OxP/R848@PLEL to 10 PM mice. Tumor regression was observed in four mice on the sixteenth day of imaging. According to the RECIST 1.1, complete remission of the tumor is defined as the complete disappearance of the tumor focus for at least 4 weeks. Therefore, we posit that the mice with no fluorescence signal reached a state of complete remission on day 44. Subsequently, the cell suspension was injected intraperitoneally to mimic tumor recurrence.

The results demonstrated that the treated mice effectively resisted the reinvasion of tumor cells (Figure 8B,C), whereas the control group mice exhibited visible metastatic foci in the abdominal cavity. Flow cytometry analysis of spleen samples revealed a significant increase in central memory T cells (Tcm cells) within the treatment group (Figure 8D; Figure S6B, Supporting Information). In summary, OxP/R848@PLEL exerts a highly effective anti‐tumor effect and establishes a stable and enduring anti‐tumor immune memory to prevent tumor recurrence.

### 2.8 Mechanisms of In Vivo Anti‐Tumor Effects

**Figure 9:**
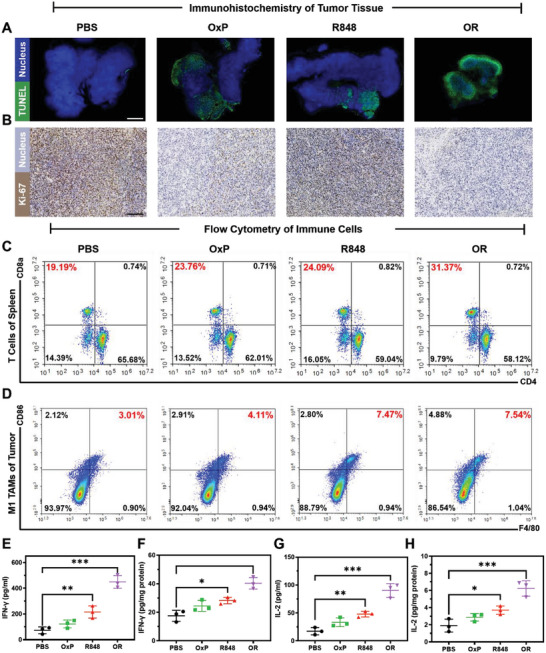
Anti‐tumor mechanism of OxP/R848 in vivo. A) TUNEL test of tumor apoptosis in each group. Scale bars: 1.25 mm. B) Proliferation of tumor detected by Ki‐67 staining. Scale bars: 150 µm. C) CD3^+^CD8^+^ T cells and CD3^+^CD4^+^ T cells in the spleen (n = 3). D) Proportion of M1‐TAMs in TME in different administration groups (n = 3). E) The level of IFN‐γ in serum of mice in each group (n = 3). F) IFN‐γ levels in the TME of mice in each group (n = 3). G) The level of IL‐2 in serum of mice in each group (n = 3). H) IL‐2 levels in the TME of mice in each group (n = 3). Data are presented as mean ± SD. *P* values were determined by Student's t‐test (*p < 0.05, **p < 0.01, ***p < 0.001).

The in vivo anti‐PM results for CRC demonstrated the synergistic effects of chemo‐immunotherapy. Based on the in vitro cytotoxicity and immune exploration results, we investigated the synergistic anti‐tumor mechanism of OxP/R848. We evaluated tumor tissue apoptosis (**Figure** **9**A) and proliferation (Figure 9B) by immunohistochemical staining. Blue fluorescence indicates the nucleus, and green fluorescence represents the fragmented DNA in TUNEL staining. In Ki‐67 staining, blue denotes the nucleus, and brown signifies Ki‐67 (a nuclear‐proliferating antigen). Following OxP/R848 treatment, marked apoptosis and a decrease in tumor cell proliferation were observed in the tumor tissue.

Flow cytometry analysis was conducted to examine the proliferation of CD8^+^ T cells in the spleen (Figure 9C) and the proportion of M1‐TAMs in the TME (Figure 9D). CD8^+^ T cells are the key executors of anti‐tumor immune responses.^[^ ^55^ ^]^ OxP or R848 alone significantly enhanced CD8^+^ T cell proliferation (*p < 0.05). Combining the two drugs further increased the proportion of CD8^+^ T cells in the spleen (Figure S8C, Supporting Information) by exerting a “tumor‐like vaccine‐like” effect. The antitumor effect of OxP/R848@PLEL was weakened (Figure S7A,B, Supporting Information) when CD8^+^ T cells were depleted using anti‐mouse CD8α (Figure S7C,D, Supporting Information), indicating that CD8^+^ T cells play a crucial role in mediating the antitumor effects of OxP/R848@PLEL. In addition, TAMs are novel therapeutic targets for malignant tumors.^[^ ^56^ ^]^ Treatment with R848 alone or combined with OxP increased the proportion of M1‐TAMs within the TME (Figure S8D, Supporting Information), promoting inflammation and exerting anti‐tumor effects.

The IFN‐γ signaling pathway can augment the immunogenicity of tumor cells, thereby leading to the upregulation of MHC I expression and the enhancement of CD8^+^ T cell activation.^[^ ^57^ ^]^ Additionally, IL‐2 can expand T cells with maintenance of functional activity.^[^ ^58^ ^]^ The levels of IFN‐γ and IL‐2 in both serum and TME were quantified by ELASA (Figure 9E–H). The results showed that the combination of OxP and R848 could increase the levels of IFN‐γ and IL‐2 in both serum and TME, which were consistent with the observed potent antitumor efficacy.

## 3 Conclusion

To sum up, we developed a novel OxP/R848@PLEL drug formulation via simple physical mixing. This system exhibited exceptional biocompatibility, injectability, and thermosensitivity while significantly prolonging the retention time of free drugs in the abdominal cavity. OxP effectively suppressed the proliferation of CRC cells and promoted the release of ICD marker molecules. The immune adjuvant R848 synergistically induced DCs maturation and activated anti‐tumor immune response. R848 also increased the proportion of anti‐tumor M1‐TAMs in the TME. In the anti‐tumor study in vivo, OxP/R848@PLEL reduced the tumor load, inhibited the production of ascites, and established long‐term immune memory to prevent tumor recurrence. Overall, the injectable thermosensitive hydrogel‐based combined chemotherapy‐immunity strategy is safe, efficient, and quality‐controllable. We have confidence that OxP/R848@PLEL is a promising therapeutic option for patients with diffuse PM in advanced CRC who are ineligible for surgical intervention.

## 4 Experimental Section

### Materials

PEG (Mn = 1500) and Sn(Oct)_2_ (95%) were procured from Sigma‐Aldrich (St. Louis, MO, USA). D, L‐LA was obtained from Daigang Chemicals (Jinan, China). OxP and R848 were supplied by Macklin Biochemical Co., Ltd. (Shanghai, China). RMPI 1640 medium, DMEM, fetal bovine serum (FBS), and penicillin‐streptomycin were purchased from Gibco, Inc. (USA). The CCK‐8 was purchased from MedChemExpress (NJ, USA). Sulfo‐Cy5.5 was provided by Beijing Fluorescence Biotechnology Co., Ltd (Beijing, China). Recombinant mouse GM‐CSF, IL‐4, and IL‐10 were purchased from Peprotech. Mouse IFN‐γ ELISA kit, Mouse IL‐2 ELISA Kit and antibodies for flow cytometry were obtained from BioLegend (USA). *d*‐Luciferin potassium salt was acquired from the Shanghai Yeasen Biotechnology Co., Ltd. (Shanghai, China). Anti‐mouse CD8α (clone 2.43) was purchased from Selleck (USA). All other chemicals used in this study were of analytical grade and used without further purification.

### Cell Culture and Animals

CT26‐luc and Raw264.7 were cultured in RPMI 1640 medium and DMEM, respectively, supplemented with 10% FBS, 1% penicillin‐streptomycin. The cultures were maintained at 37 °C under a relative humidity of 95%.

BALB/c mice (female, 20 ± 2 g) aged 6—8 weeks were purchased from Gempharmatech Co., Ltd (Jiangsu, China). The mice were housed under specific pathogen‐free conditions and provided ad libitum access to food and water. All animal experiments were approved by the Experimental Animal Ethics Committee of the State Key Laboratory of Biotherapy at Sichuan University (No: 20 220 531 049).

### Synthesis and Structural Characterization of PLEL Block Copolymers

Triblock copolymer PLEL was synthesized via ring‐opening polymerization of PEG and D,L‐LA catalyzed by Sn(Oct)_2_.^[^ ^27^ ^]^ A dry three‐neck flask was filled with PEG, D, L‐LA, and Sn(Oct)_2_ (0.3% of the total weight of the raw materials), followed by vacuum pumping to remove water. Subsequently, the reaction mixture was heated to 140 °C in an oil bath under a nitrogen atmosphere for 10 h. The crude product obtained was dissolved in ultra‐pure water at RT, followed by heating to 80 °C for precipitation of the target product and removal of unreacted monomers and low molecular weight products. The precipitate was freeze‐dried until a constant weight was obtained. The structure and molecular weight of PLEL copolymers were characterized by ^1^H‐NMR (Varian 400 spectrometer, Varian, USA) and gel permeation chromatography (GPC‐20A, Japan).

### Preparation and Properties of OxP/R848@PLEL Potion‐Containing Gel

First, the PLEL block copolymer was dissolved in PBS (pH 8.0) at RT to prepare blank PLEL micelles at 20 wt%. Second, OxP/R848@PLEL was obtained by mixing a certain amount of OxP and R848 (OxP: 0.6 mg mL^−1^, R848: 0.1 mg mL^−1^) with a PLEL micellar solution at RT. The drug‐loaded hydrogel was sterilized using a 0.22 µm filter membrane and stored at 4 °C for later use.

Subsequently, the thermo‐sensitive phase transformation behavior of the OxP/R848@PLEL using the inverted tube method was investigated. Specifically, 1 mL of hydrogel was added to a vial with an inner diameter of 1 cm and heated from RT to 37 °C before being inverted. If no considerable flow occurred within 1 min, a sol‐gel transition occurred. To demonstrate the injectability and in vivo gelation of hydrogels, 200 µL of drug‐loaded hydrogel was injected into the peritoneal cavity of mice. After 10‐min of incubation, the mice were euthanized under isoflurane anesthesia, and the gelation process was observed.

The mechanical properties of the drug‐loaded hydrogels with temperatures were investigated using a rotating rheometer (HAAKE Mars III, Thermo Scientific, USA). The sample precooled at 4 °C was placed on a circular flat fixture with a diameter of 20 mm. Low‐viscosity silicone oil was applied to the edge of the sample to prevent moisture volatilization owing to the rising temperature. The sample's temperature was increased from 10 to 60 °C at the rate of 1 °C min^−1^, while maintaining a stress of 4.0 dyn cm^−2^ and frequency of 1.0 Hz. Data were collected to determine the changes in the energy G′ and G″ of the samples with respect to temperature. In addition, the size and shape of the OxP/R848@PLEL hydrogel in different temperatures were validated by TEM.

### Drug Release In Vitro

The release behavior of OxP and R848 from the PLEL hydrogel was investigated using an improved dialysis method.^[^ ^59^ ^]^ One milliliter of drug‐containing PBS solution or drug‐loaded PLEL hydrogel (OxP: 0.6 mg mL^−1^, R848: 0.1 mg mL^−1^) was placed into a dialysis bag and incubated at 37 °C for 30 min. Then the dialysis bags were transferred to centrifuge tubes containing 10 mL of pH 7.4 PBS and incubated on a shaker at 100 rpm and 37 °C. The release medium was extracted at predetermined time points, followed by adding an equal volume of preheated PBS. The collected release medium was stored at −20 °C and subsequently analyzed for drug release using high‐performance liquid chromatography.

### Drug Retention In Vivo

To evaluate the sustained‐release performance of the PLEL hydrogel as a drug delivery carrier, the fluorescent dye Sulfo‐Cy5.5 as a model drug to investigate drug retention in the abdominal cavity was utilized . BALB/c mice were intraperitoneally injected with 200 µL PBS solution containing Sulfo‐Cy5.5 or PLEL hydrogel loaded with Sulfo‐Cy5.5. To visualize the in vivo behavior of the drug, fluorescence images were acquired using an IVIS. The fluorescence intensity data were recorded using the LivingImage software for subsequent image analysis.

### In Vitro Cytotoxicity Test

The cytotoxicity of free OxP and R848 in CT26‐luc cells using a CCK‐8 assay was first evaluated. The cells were inoculated in 96‐well plates with 100 µL complete medium per well and incubated overnight. The original medium was replaced with a fresh complete medium or varying concentration of OxP or R848. After treatment for 24, 48, and 72 h, the cells were subjected to CCK‐8 assay and the absorbance was measured at 450 nm using a Bio‐Rad instrument. The relative cell activity in the experimental group was expressed as a percentage of that in the control group.

Next, the cytotoxicity of OxP@PLEL against CT26‐luc cells using a transwell co‐culture system was assessed. CT26‐luc cells were seeded into 24‐well plates and incubated overnight. Subsequently, 100 µL of PBS and free OxP were added directly to the upper chamber of the transwell apparatus. Currently, 100 µL of blank PLEL hydrogel or OxP@PLEL was added to the same upper chamber and left for 30 min to solidify from sol phase into gel phase before insertion into its corresponding position within the co‐culture system. The co‐culture system was incubated and refreshed with 500 µL preheated fresh complete medium every 24 h. After culturing for 24, 48, and 72 h, CCK‐8 treatment was administered to the cells, and the absorbance at 450 nm was measured. The relative cell activity in the experimental group was calculated as a percentage of that in the control group.

### Evaluation of ICD Effect

CRT expression was detected through immunofluorescence. CT26‐luc cells were inoculated onto polylysine‐coated coverslips and incubated for 12 h before treatment with fresh medium containing varying concentrations of OxP (OxP‐L: 2.5, OxP‐M: 10, OxP‐H: 50 µg mL^−1^) for an additional 6 h. The culture medium was collected, and the cells were fixed in 4% paraformaldehyde at RT for 15 min. After fixation, the samples were sealed with a closed buffer solution for 60 min. The diluted CRT primary antibody was added and incubated overnight at 4 °C. Subsequently, a fluorescein‐conjugated secondary antibody was added and incubated in the dark at RT for 2 h. Finally, the nuclei were stained with hoechst33342. The slides were sealed with an anti‐fluorescence attenuation tablet and immediately observed under a fluorescence microscope. Three visual fields with a cell count >50 were randomly selected for each slide, and the percentage of CRT^+^ cells was calculated.

Secretion of HMGB1 and ATP into the cell culture medium was quantified by ELISA. The collected cell supernatant was centrifuged at 3000 rpm for 10 min to eliminate particles and polymers. According to the manufacturer's instructions, mouse HMGB1 and ATP ELISA kits were used to determine the concentrations of HMGB1 and ATP in the cell culture, respectively.

### BMDC Maturation Assay

BMDCS were isolated from female C57BL/6 mice using a method described previously.^[^ ^60^ ^]^ Mice were anesthetized and sterilized with 75% ethanol before being transferred to a super‐clean table. The femurs and tibias of mice were carefully extracted. The two ends were cut with scissors, and the bone cavity was rinsed with PBS. Bone marrow cells were collected by a 70 µm filter, followed by the removal of red blood cells using red blood cell lysate. The cells were cultured in RPMI‐1640 complete medium supplemented with 10% FBS, 1% penicillin‐streptomycin, 20 ng mL^−1^ mouse recombinant GM‐CSF, and 10 ng mL^−1^ mouse recombinant IL‐4. Half of the culture medium was replaced every 2 days. After 7 days, non‐adherent and loosely adherent cells were harvested for further use.

CT26‐luc cells were seeded in 12‐well plates and cultured for 12 h. The original medium was replaced with fresh medium containing PBS, OxP, R848, or OxP/R848 and incubated at 37 °C for 24 h. Subsequently, the collected BMDCs were co‐cultured with treated cancer cells. After incubation for 24 h, BMDCs from each group were collected and incubated with FITC anti‐mouse CD11c, APC anti‐mouse CD86, and PE anti‐mouse CD80 on ice for 30 min. After washing and suspension in PBS, flow cytometry was performed for detection.

### TAMs Polarization

Raw264.7 cells were inoculated in 12‐well plates and allowed to adhere before treatment with fresh medium containing PBS, OxP, R848, or OxP/R848 for 24 h. Raw264.7 of each group were collected and incubated with FITC anti‐mouse F4/80 and PE anti‐mouse CD86 on ice for 30 min. Cells from each group were harvested and incubated with FITC anti‐mouse F4/80 and PE anti‐mouse CD86 antibodies on ice for 30 min. After washing and suspension in PBS, flow cytometry was performed for detection.

### Co‐Incubation of BMDMs and CT26‐Luc Cells

Bone marrow cells were isolated from the C57BL/6 mice and cultured in DMEM complete medium containing M‐CSF (10 ng mL^−1^) to induce bone marrow cells to differentiate into macrophages (BMDMs). The BMDMs were treated with PBS, R848, or IL‐4&IL‐10 to induce the polarization of BMDMs into M0, M1, and M2 states, respectively. Subsequently, a transwell co‐culture system (Figure S3C, Supporting Information), incubated CT26‐luc cells with BMDMs, and detected the proliferation of cancer cells by CCK‐8 assay was constructed.

### In Vivo Drug Dose Screening

An acute toxicity test of OxP@PLEL was conducted on 15 healthy BALB/c mice acclimated for 1 week. The mice were randomly divided into five groups of three animals each. Each group received a single intraperitoneal injection of either 200 µL PBS or OxP@PLEL (OxP: 8, 16, 24, and 32 mg kg^−1^). The weight and daily observations of the mice's state (hair condition, diet intake, and defecation) and survival were recorded.

Subsequently, OxP@PLEL were repeatedly administered to 25 healthy BALB/c mice. Then subjects were randomly allocated into five groups and administered intraperitoneal injections of 200 µL PBS or OxP@PLEL (OxP: 4, 6, 8, and 12 mg kg^−1^) per group. The drug was administered at 4‐day intervals for three doses, and the weights of the mice were measured twice daily. Daily records of hair, diet, defecation, and survival rate were kept.

In the two‐drug combination dose screening, the dose of OxP was determined based on the results of toxicity experiments, whereas the dose of R848 depended on its solubility in PLEL micelles. To establish the PM model of CRC, BALB/c mice were injected with CT26‐luc cells (8 × 10^5^ cells per mouse) intraperitoneally. Four days after inoculation, live imaging was performed on small animals. Mice with low or no fluorescence signals were excluded from the study. The successfully modeled mice were randomly divided into seven groups, each consisting of four mice. Each group was intraperitoneally injected 200 µL of PBS, OxP@PLEL (OXP: 4, 6 mg kg^−1^) or OxP/R848@PLEL (OxP/R848: 4, 0.5; 4, 1; 6, 0.5; 6, 1 mg kg^−1^). The drug was administered at 4‐day intervals for three doses. Mouse weight was measured twice daily, and the status (hair, diet, and defecation) and survival were recorded daily. On the sixteenth day post‐inoculation, the mice were euthanized under isoflurane anesthesia. Abdominal tumors were excised and weighed, and the peritoneal fluid volume was measured.

### Evaluation of the Antitumor Effect of OxP/R848@PLEL In Vivo

Mice with PM were randomly assigned to each of the eight groups. Each group received an intraperitoneal injection of 200 µL of either PBS, blank PLEL, OxP@PBS, R848@PBS, OxP/R848@PBS, OxP@PLEL, R848@PLEL or OxP/R848 @PLEL (OxP: 6 and R848: 1 mg kg^−1^). The medication was administered at 4‐day intervals for three doses. The mice were weighed twice daily. On day 16, five mice from each group were euthanized under isoflurane anesthesia for the surgical extraction of abdominal tumors and major organs, and ascites volumes were recorded. The remaining 10 mice from each group were observed for survival.

### Evaluation of Anti‐Tumor Immune Memory of OxP/R848@PLEL

Ten PM mice were intraperitoneally injected with 200 µL of OxP/R848@PLEL (OxP: 6 and R848: 1 mg kg^−1^) every 4 days for three doses. Mice exhibiting no fluorescence signal on in vivo imaging on days 16 and 44 were considered to have achieved complete tumor regression. On the same day, CT26‐luc cells (8 × 10^5^ cells per mouse) were intraperitoneally injected into both mice with tumor‐regressed and untreated mice to simulate residual tumor cells. On days 48 and 54, tumor growth was observed using an IVIS. On day 54, the mice were euthanized with isoflurane anesthesia and underwent surgical removal of the abdominal tumors and spleens. The central memory T cell content in the spleen was analyzed using flow cytometry.

### Anti‐Tumor Mechanism of OxP/R848@PLEL

Mice with PM were randomly divided into four groups of six mice per group. Each group was intraperitoneally injected with 200 µL of PBS, OxP@PLEL, R848@PLEL, or OxP/R848@PLEL (OxP: 6 and R848: 1 mg kg^−1^). The medication was administered at 4‐day intervals for three doses. On day 16, the mice were euthanized under isoflurane anesthesia, and their abdominal tumors and spleens were surgically excised. Proliferation and apoptosis of tumor tissues were analyzed by immunohistochemical staining, while T cell activation in the spleen and the proportion of M1‐TAMs in the TME were assessed via flow cytometry. In addition, the levels of IFN‐γ and IL‐2 in the serum and TME of mice in each group were quantified by ELISA. In order to prove the role of CD8^+^ T cells in OxP/R848@PLEL anti‐tumor therapy, OxP/R848@PLEL &Anti‐CD8α group was set up in addition to the model group and OxP/R848@PLEL treatment group. The biological activity of CD8^+^ T cells were restricted by binding to mouse CD8α (100 µg per mouse, once every four days, for a total of three times).

### Statistical Analysis

Data were obtained from three independent experiments and analyzed using GraphPad Prism 8.0. All date were presented as the mean ± SD. The *P* values for survival curves were determined by the Gehan‐Breslow‐Wilcoxon test, while Student's *t*‐test was employed for comparing between two groups. For statistical analysis among multiple groups, one‐way ANOVA with Tukey's test was utilized. Statistical significance was defined as ^*^p < 0.05, while ^**^p < 0.01, and ^***^p < 0.001 indicated extreme significance.

## Conflict of Interest

The authors declare no conflict of interest.

## Supporting information

Supporting Information

## Data Availability

The data that support the findings of this study are available from the corresponding author upon reasonable request.
