# Supplementary material for: Enhanced Chemo‐Immunotherapy Strategy Utilizing Injectable Thermosensitive Hydrogel for The Treatment of Diffuse Peritoneal Metastasis in Advanced Colorectal Cancer
Source: Adv Sci (Weinh). 2023 Oct 24;10(35):2303819. doi: 10.1002/advs.202303819 (PMC10724414; doi:10.1002/advs.202303819)
Supplement: Supplementary file 1 — Supporting Information [file ADVS-10-2303819-s001.pdf]

ADVANCED SCIENCE
Open Access

Supporting Information

for *Adv. Sci.*, DOI 10.1002/advs.202303819

Enhanced Chemo-Immunotherapy Strategy Utilizing Injectable Thermosensitive Hydrogel for The Treatment of Diffuse Peritoneal Metastasis in Advanced Colorectal Cancer

*Meng Wang, DanRong Hu, Yun Yang, Kun Shi, JiaNan Li, QingYa Liu, YiCong Li, Ran Li, Meng Pan, Dong Mo, Wen Chen, XiCheng Li and ZhiYong Qian**

Supporting Information

Enhanced Chemo-immunotherapy Strategy Utilizing Injectable Thermosensitive Hydrogel for the Treatment of Advanced Diffuse Peritoneal Metastatic Colorectal Cancer

*Meng Wang, DanRong Hu, Yun Yang, Kun Shi, JiaNan Li, QingYa Liu, YiCong Li, Ran Li, Meng Pan, Dong Mo, Wen Chen, XiCheng Li, and ZhiYong Qian**

M. Wang, Dr. Y. Yang, Dr. K. Shi, J. Li, Dr. Q. Liu, Y. Li, M. Pan, D. Mo, W. Chen, X. Li, Prof. Z. Qian
Department of Biotherapy
Cancer Center and State Key Laboratory of Biotherapy
West China Hospital
Sichuan University
Chengdu ,610041, China
E-mail: anderson-qian@163.com

Dr. D. Hu, R. Li
Rehabilitation Medicine Center and Institute of Rehabilitation Medicine
Key Laboratory of Rehabilitation Medicine in Sichuan Province
West China Hospital
Sichuan University
Chengdu, 610041, China

Keywords: colorectal cancer, diffuse peritoneal metastasis, thermosensitive hydrogel, chemo-immunotherapy, intraperitoneal chemotherapy

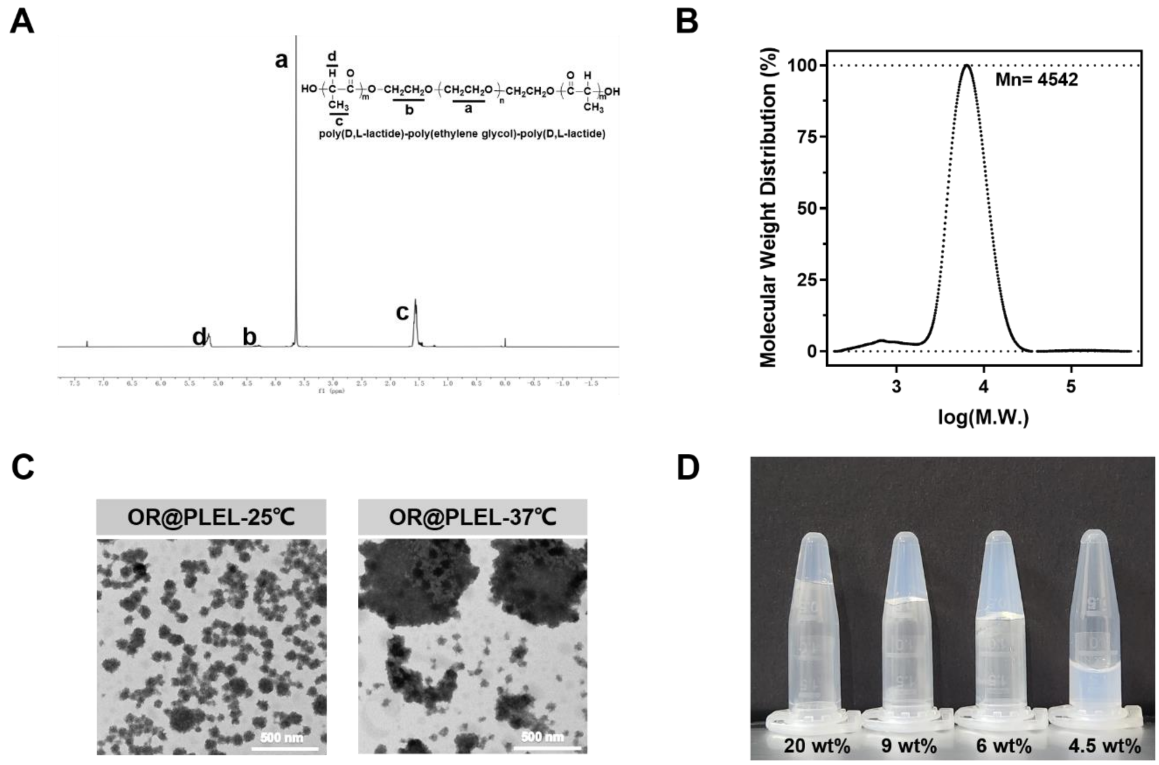

Figure S1. Structural characterization and properties of PLEL hydrogels. A) ^{1}H NMR spectrum of PLEL (solvent: $CDCl_3$). B) The molecular weight distribution of PLEL triblock copolymers. C) The TEM images of OR/PLEL micelles at 25°C and 37°C. D) The 20 wt% concentration of the PLEL micelle solution was diluted to different levels to evaluate its gelation capacity.

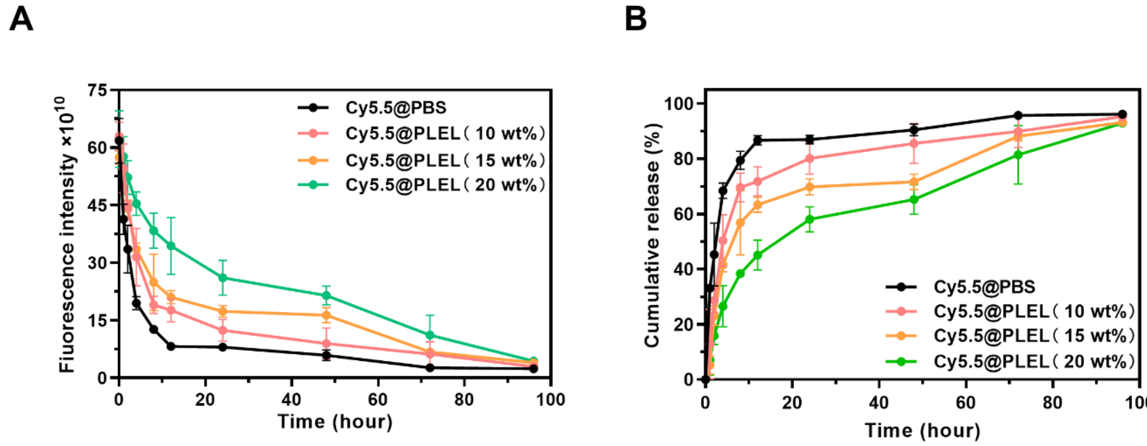

Figure S2. In vivo retention of drug-containing PLEL hydrogel. A) Quantitative analysis of Cy5.5 fluorescence intensity in different media over time (n = 3). B) Quantitative analysis of in vivo release of Cy5.5 in different release media over time (n = 3). Data are presented as mean ± SD.

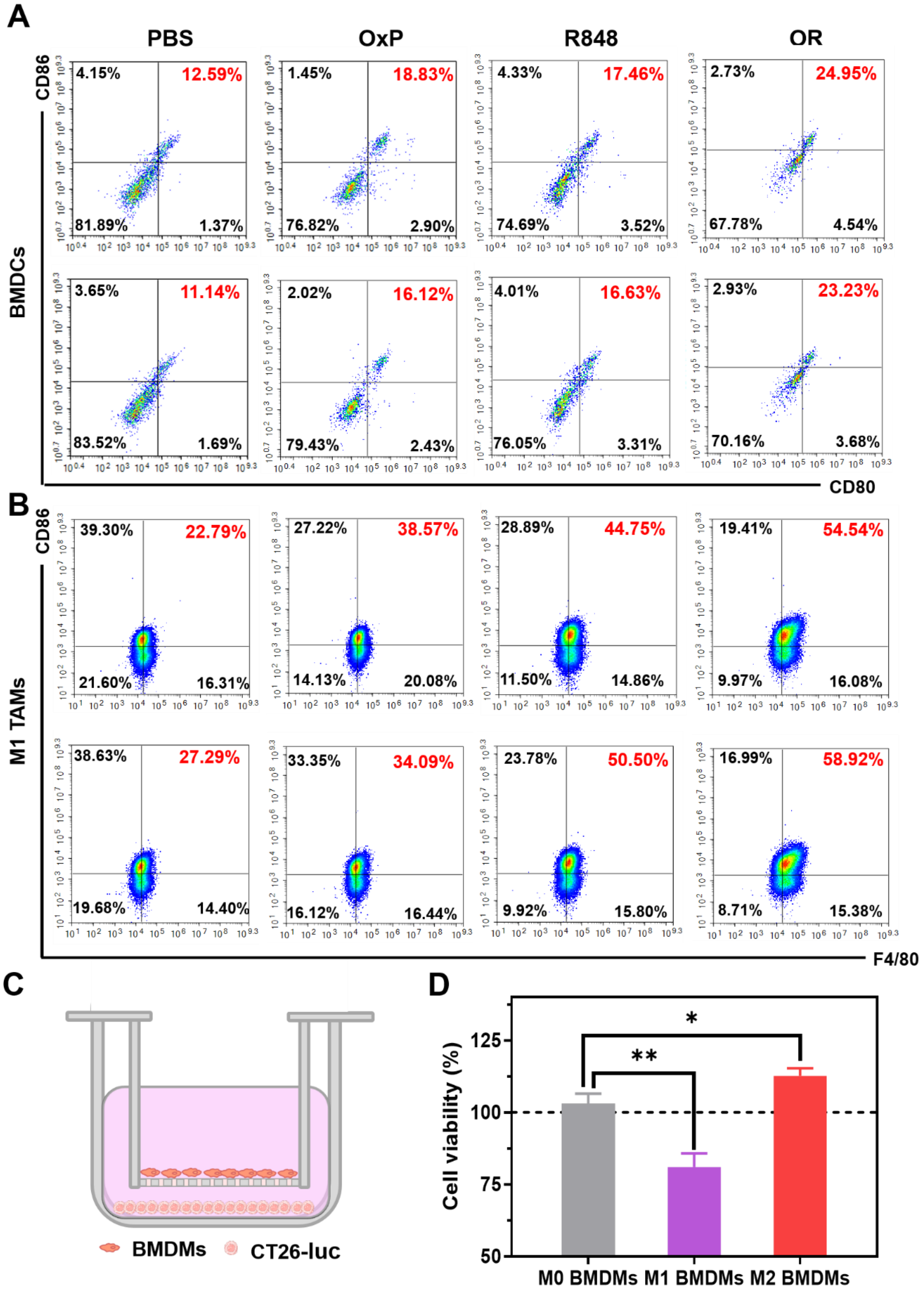

Figure S3. Immunological analysis of OxP and R848. A) Flow cytometry of CD80 and CD86 on the BMDCs (n = 3). B) Proportion of M1 phenotype Raw264.7 cells after different

treatments (n = 3). C) Schematic diagram depicting the construction of a transwell system for co-incubation of BMDMs and CT26-luc cells. D) Cell viability of CT26-luc cells (n = 3). Data are presented as mean ± SD. *P* values were determined by Student's *t*-test (*$p < 0.05$, **$p < 0.01$).

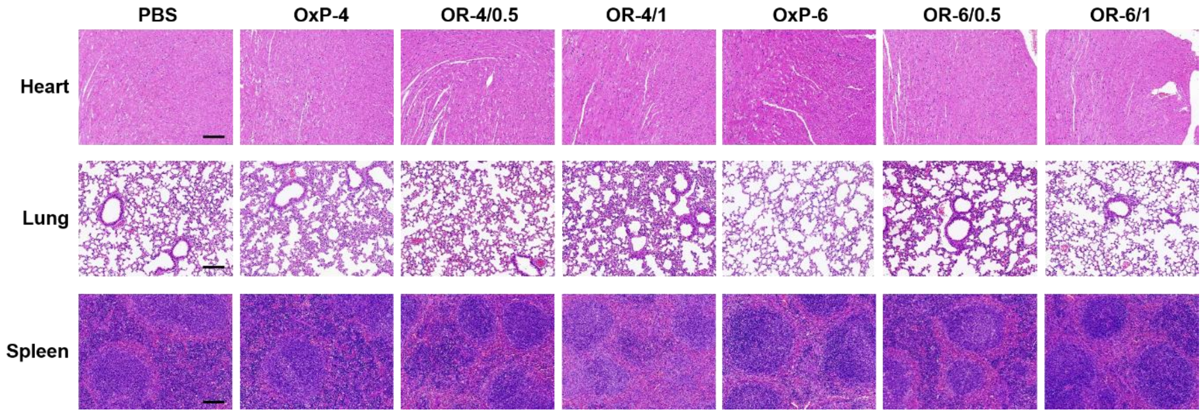

Figure S4. Dose optimization of OxP/R848@PLEL in PM treatment. H&E staining of heart, lung, and spleen tissues of each group. Scale bars: 200 μm.

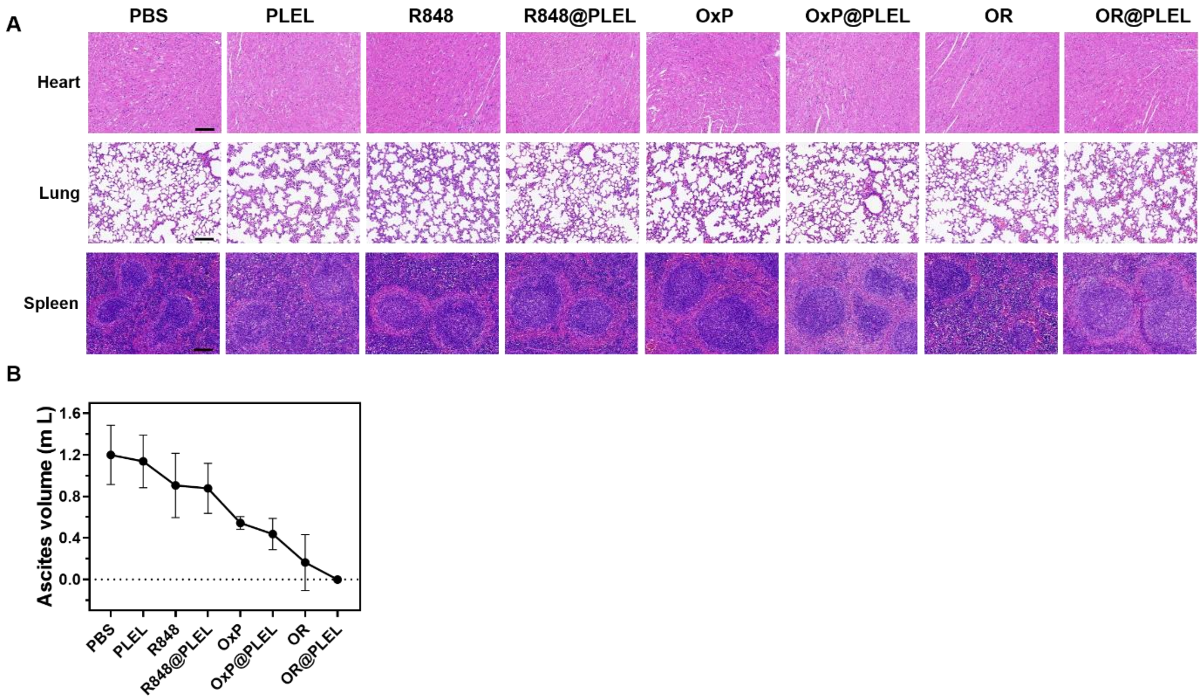

Figure S5. In vivo pharmacodynamic study of OxP/R848@PLEL in PM treatment. A) H&E staining of heart, lung, and spleen tissues of each group. Scale bars: 200 μm. B) Ascites volume of mice in each group (n = 5). Data are presented as mean ± SD.

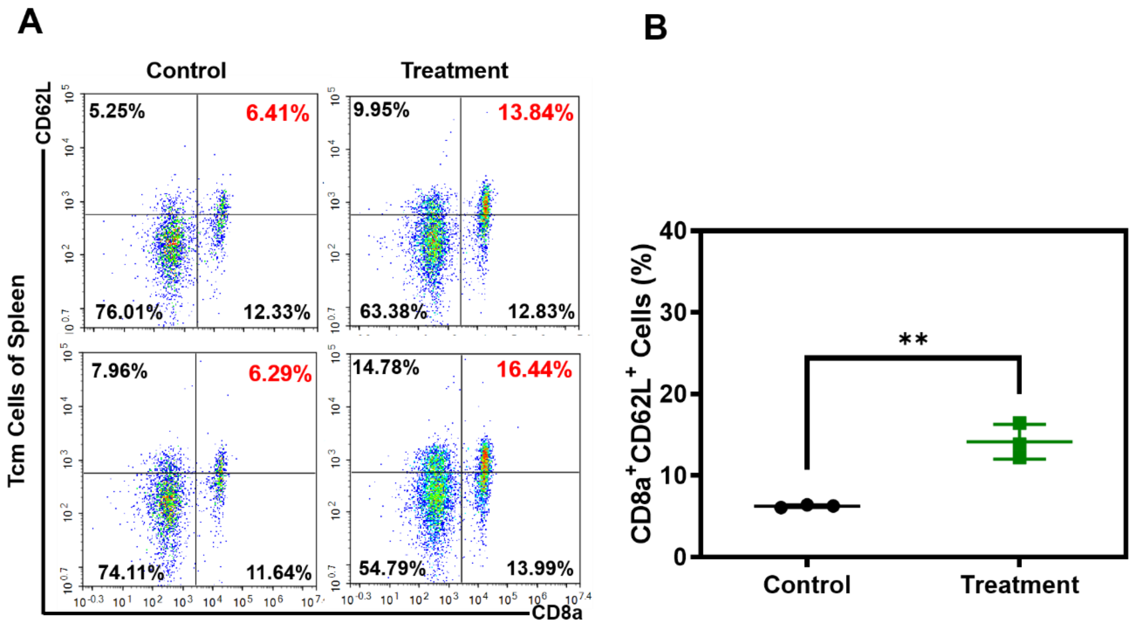

Figure S6. A, B) Flow cytometry of central memory T cells in the spleen (n = 3). Data are presented as mean ± SD. *P* values were determined by Student's *t*-test (**$p < 0.01$).

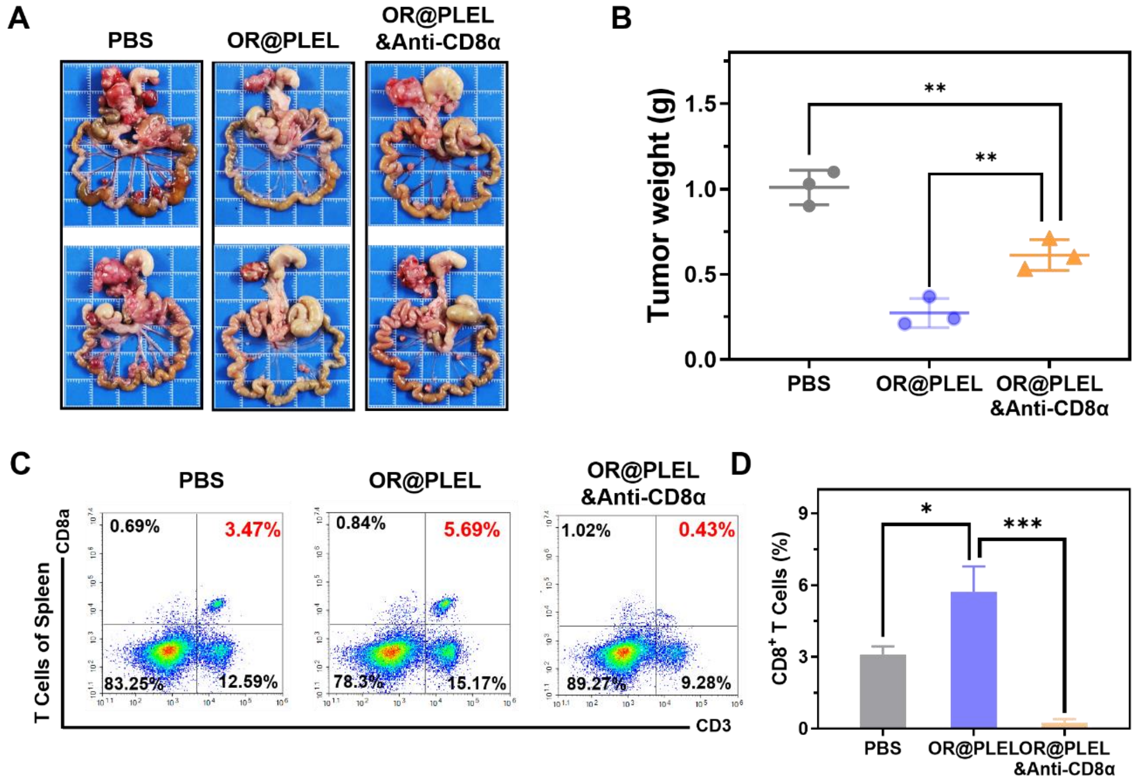

Figure S7. Effect of $CD8^+$ T cell depletion on the antitumor effect of OxP/R848@PLEL. A) Photographs of PM foci of CRC. B) Tumor weight of mice in each group (n = 3). C) Flow

cytometry of CD3$^+$CD8$^+$ T cells in the spleen (n = 3). D) Proportion of CD8$^+$ T cells in the spleen (n = 3). Data are presented as mean ± SD. *P* values were determined by Student's *t*-test (*$p < 0.05$, **$p < 0.01$, ***$p < 0.001$).

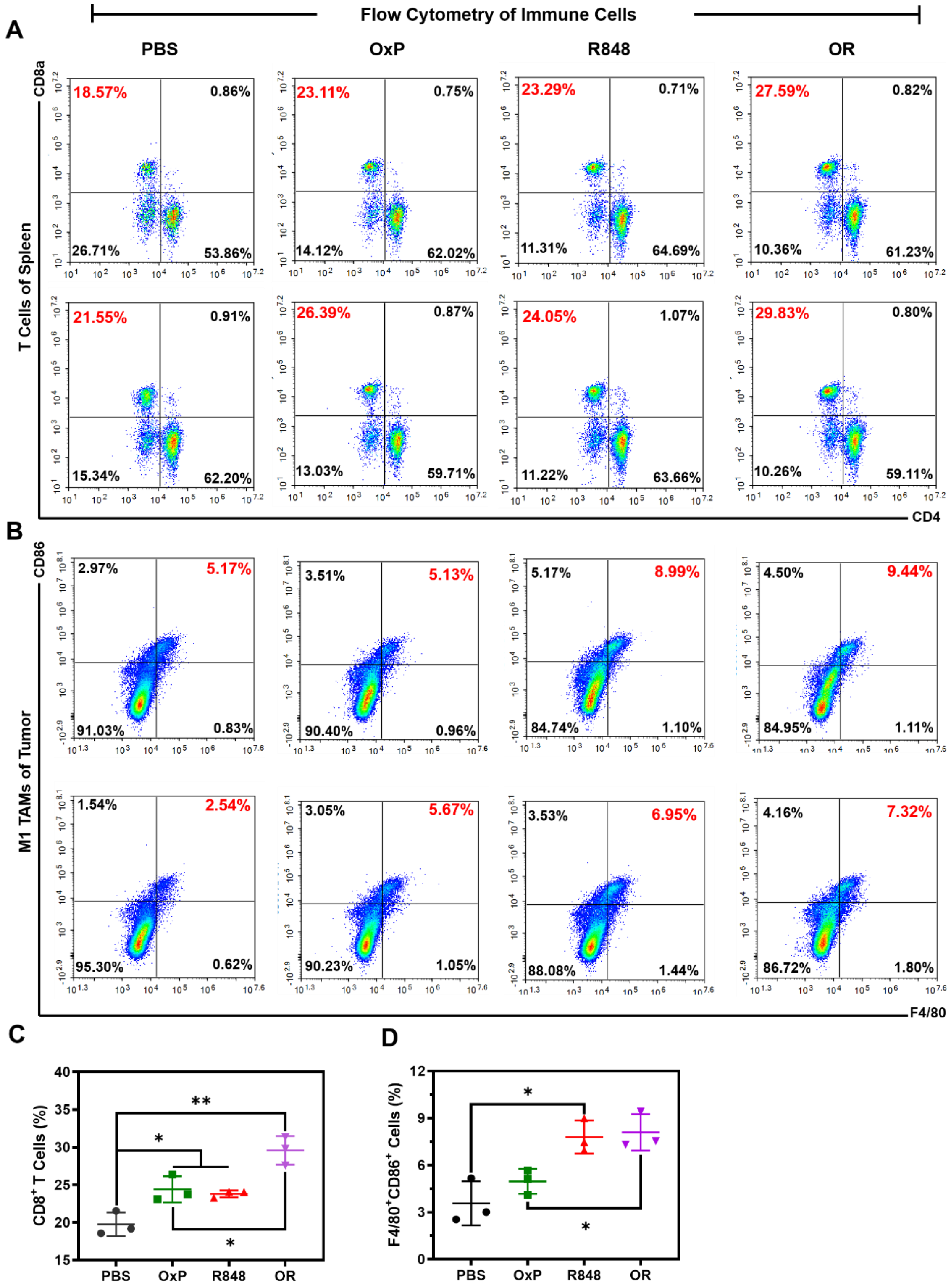

Figure S8. Anti-tumor mechanism of OxP/R848 in vivo. A) $CD3^+CD8^+$ T cells and $CD3^+CD4^+$ T cells in the spleen (n = 3). B) Proportion of M1-TAMs in TME in different administration groups (n = 3). C) Proportion of $CD8^+$ T cells in the spleen (n = 3). D) Proportion of M1-TAMs cells in the TME (n = 3). Data are presented as mean ± SD. *P* values were determined by Student's *t*-test (*$p < 0.05$, **$p < 0.01$).
